# Ba(OH)_2_ Equilibria in the System Ba-O-H-F, With Application to the Formation of Ba_2_YCu_3_O_6.5 + x_ From BaF_2_-Precursors

**DOI:** 10.6028/jres.110.011

**Published:** 2005-04-01

**Authors:** L. P. Cook, W. Wong-Ng, R. Feenstra

**Affiliations:** National Institute of Standards and Technology, Gaithersburg, MD 20899-8520; Condensed Matter Sciences Division, Oak Ridge National Laboratory, Oak Ridge, TN 37831

**Keywords:** Ba_2_YCu_3_O_6.5 +_*_x_*, BaF_2_, Ba(OH)_2_, *ex situ* process, liquid, phase equilibria, *P*_H_2_O_, pHF, processing, superconductor, thermodynamic calculations

## Abstract

The *ex situ* process for fabricating Ba_2_YCu_3_O_6.5 +_ *_x_* superconducting tapes from BaF_2_- based precursors involves a hydration/oxidation reaction at ≈730 °C to 750 °C generally written as:

$$\begin{array}{l} \left(2\text{BaF}2+Y+3\text{Cu}\right)\left(\text{amorphous}\right)+\\ \left(2H_{2}O+2.25O_{2}\right)\left(g\right)\to \\ {\text{Ba}}_{2}{\text{YCu}}_{3}O_{6.5+x}\left(s\right)+4\text{HF}\left(g\right). \end{array}$$

However, microscopic observations of partially processed films suggest the presence of a transient liquid phase during conversion. Alternatively, the conversion reaction can be rewritten as the sum of several intermediate steps, including the formation of a barium hydroxide liquid:

$$\begin{array}{l} \left({\text{BaF}}_{2}\right)\left(\text{amorphous}\right)+2H_{2}O\left(g\right)\to \\ \text{Ba}{\left(\text{OH}\right)}_{2}\left(\text{liq}\right)+2\text{HF}\left(g\right). \end{array}$$

To evaluate the possibility of a hydroxide liquid conversion step, thermodynamic calculations on the stability of Ba(OH)_2_(liq) have been completed from 500 °C to 900 °C at 0.1 MPa *p*_total_. Based on currently available data, the calculated phase diagrams suggest that a viable hydroxide reaction path exists in the higher part of this temperature range. The calculations indicate that Ba(OH)_2_(liq) may be stable at log $p_{H_{2}O}$ (Pa) values from ≈4 to 5, provided log *p*_HF_ (Pa) values can be maintained below 0 to −1. Limited experimental confirmation is provided by results of an experiment on BaF_2_(s) at 815 °C, 0.1 MPa $p_{H_{2}O}$, in which essentially all F at the surface was replaced by O. It is therefore possible that processing routes exist for producing Ba_2_YCu_3_O_6.5 +_ *_x_* based on the presence of a Ba(OH)_2_ liquid, which might have an effect on conversion rates and texturing in the superconducting film.

## 1. Introduction

**Fig. 1:**
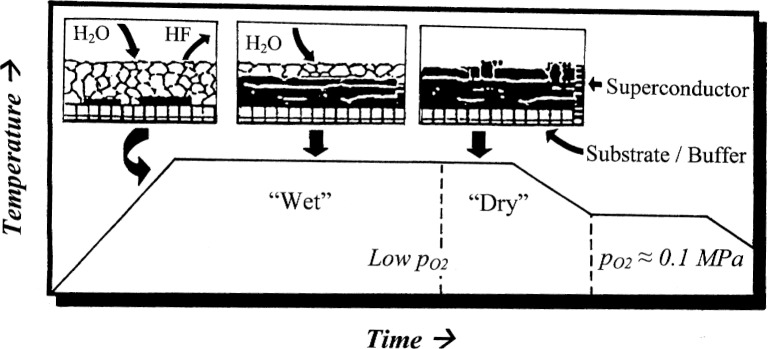
Schematic illustration of the “BaF_2_ *ex situ*” process for fabrication of Ba_2_YCu_3_O_6.5 +_ *_x_* coated conductors, showing conversion of precursor film (top), as deposited on substrate/buffer layer. Precursor is converted starting at buffer/precursor interface by reaction with water vapor entering from top. Superconductor layer (dark) grows and is then annealed at lower temperatures under dry conditions in oxygen.

The development of superconducting Ba_2_YCu_3_O_6.5 +_ *_x_*-based tape is continuing at an accelerated pace, with the promise of substantial cost-reductions and performance enhancements in a variety of high temperature superconductor (high *T*_c_) applications, such as transformer-, magnet-, and motor-windings and power transmission cables. This technology, the “second generation” of high *T*_c_ materials, depends upon the fabrication of superconducting layers deposited on flexible metallic substrates [1–4]. Generally the metallic substrates require deposition of intermediate buffer layers to serve as diffusion barriers at the metal interface, while promoting textured growth of the overlying oxide superconductor phase. One of the leading processes for producing highly textured second generation “coated conductors” is the “BaF_2_ *ex situ*” method [5–7], a process which consists of two principal steps. In the first step, amorphous films having (2 BaF_2_ + Y + 3 Cu) stoichiometry are deposited on the buffer layer by physical vapor deposition (PVD) or metal/organic deposition (MOD) techniques. The second step (shown diagrammatically in Fig. 1) involves the conversion of the amorphous film to superconducting material, nominally according to the following overall reaction:

$$\begin{array}{l} \left(2{\text{BaF}}_{2}+Y+3\text{Cu}\right)\left(\text{amorphous}\right)\\ +\left(2H_{2}O+2.25O_{2}\right)\left(g\right)\to \\ {\text{Ba}}_{2}{\text{YCu}}_{3}O_{6.5+x}\left(s\right)+4\text{HF}\left(g\right). \end{array}$$ (1)

Typically, the reaction takes place at ≈730 °C to 750 °C. The principal advantages of the *ex situ* process are that the first step is a relatively simple, rapid operation completed without the necessity of substrate heating, and that the second, more time-consuming step can be completed separately from the deposition process, without the need for vacuum conditions. The exact nature of the conversion process remains controversial. Indirect evidence has been presented for the formation of an amorphous intermediate phase (possibly a liquid) during the conversion [8].

## 2. Previous Work

Research has previously been conducted in our laboratory to determine the nature of low-melting-temperature liquids involved in the BaF_2_ *ex situ* conversion [9–11]. The relevant phase equilibria can be discussed with reference to the Ba,Y,Cu//O,F quaternary reciprocal system, portrayed in Fig. 2a as a trigonal prism in compositional phase space. Initially, the relative thermodynamic stabilities of Table 1 were used to subdivide the trigonal prism of Fig. 2a into its constituent tetrahedra, shown in Fig. 2b. The phase stabilities defining these tetrahedra have since been confirmed experimentally, and hence the tetrahedra serve as a valid outline for discussion of the conversion process. In traversing along a compositional vector from the oxides at the base of the trigonal prism, to the fluorides at the top, the three constituent tetrahedra encountered are: BaF_2_-BaO-½Y_2_O_3_-CuO*_x_*, BaF_2_-YF_3_-½Y_2_O_3_-CuO*_x_*, and BaF_2_-YF_3_-CuF_2_-CuO*_x_*. The first two tetrahedra share the very stable BaF_2_-½Y_2_O_3_-CuO*_x_* compositional plane, where the ideal compositions of PVD precursor films would be plotted. In terms of fluoride/oxide ratio, the progression of such films during *ex situ* conversion to superconductor would ideally follow the path shown in the lower tetrahedron of Fig. 2b. Yet, melting temperatures below 815 °C were not found in this tetrahedron, even in the presence of H_2_O-containing gas mixtures with reduced $p_{O_{2}}$. Locally, it is possible that PVD compositions might deviate from the BaF_2_-½Y_2_O_3_-CuO plane in the fluoride-direction. Also, MOD films initially have compositions with more fluoride than would correspond to compositions in this plane. Therefore our search for low-melting liquids was also extended into the fluoride-rich regions of Fig. 2. Our experimental investigation of the BaF_2_-YF_3_-½Y_2_O_3_-CuO*_x_* tetrahedron has shown that melting temperatures in that tetrahedron are in excess of 800 °C, even in the presence of H_2_O, or with reduced $p_{O_{2}}$.

In the BaF_2_-YF_3_-CuF_2_-CuO*_x_* tetrahedron, fluoride-rich liquids melting as low as 580 °C were found; possibly these could be involved in the early stages of MOD film conversion. However, for low-melting liquids to be generally operative in conversion of both PVD and MOD films, they would need to lie closer in phase space to the BaF_2_-½Y_2_O_3_-CuO*_x_* plane. Consequently, our search for low-melting liquids has turned to the possible presence of Ba(OH)_2_, a compound known to melt much lower (at ≈ 408 °C) [12] than the other phases and components of the Ba,Y,Cu//O,F system. Under suitable $p_{H_{2}O}$ and temperature, Ba(OH)_2_ could proxy for the BaO component.

## 3. Possible Presence of Ba(OH)_2_ During *Ex Situ* Conversion

Reaction (1) may be rewritten as the sum of several constituent reactions, based in part on high temperature powder x-ray diffraction (HTXRD) observations in our laboratory [10,13] and in other laboratories [14,15]. Moreover, under sufficiently high $p_{H_{2}O}$, the BaO component of Fig. 2 could effectively be replaced by Ba(OH)_2_, resulting in the following conversion steps:

$$3\text{Cu}\left(\text{amorphous}\right)+1.5O_{2}\left(g\right)\to 3\text{CuO}\left(s\right)$$ (2)

$$\begin{array}{l} Y\left(\text{amorphous}\right)+\text{CuO}\left(s\right)+0.75O_{2}\left(g\right)\to \\ 0.5Y_{2}{\text{Cu}}_{2}O_{5}\left(s\right) \end{array}$$ (3)

$$2{\text{BaF}}_{2}\left(\text{amorphous}\right)\to 2{\text{BaF}}_{2}\left(s\right)$$ (4)

$$\begin{array}{l} 2{\text{BaF}}_{2}\left(s\right)+4H_{2}O\left(g\right)\to 2\text{Ba}{\left(\text{OH}\right)}_{2}\left(\text{liq}\right)\\ \;+4\text{HF}\left(g\right) \end{array}$$ (5)

$$\begin{array}{l} 2\text{Ba}{\left(\text{OH}\right)}_{2}\left(\text{liq}\right)+0.5Y_{2}{\text{Cu}}_{2}O_{5}\left(s\right)+2\text{CuO}\left(s\right)\\ \to {\text{Ba}}_{2}{\text{YCu}}_{3}O_{6.5+x}\left(s\right)+2H_{2}O\left(g\right). \end{array}$$ (6)

Reactions (5) and (6) indicate the presence of Ba(OH)_2_ liquid as an intermediate step.

## 4. Goal and Approach of Present Investigation

The primary goal of this paper is to evaluate the possible presence of Ba(OH)_2_ liquid during the *ex situ* conversion process, using a combination of experimental and calculative methods. Demonstrating the presence of liquids at high temperature is of course is not possible by HTXRD, and so we must rely on less direct methods. Also, maintaining the high $p_{H_{2}O}$ and low *p*_HF_ thought necessary to form Ba(OH)_2_ liquids from BaF_2_ at high temperature in a controlled experimental environment is in itself a daunting task. For this purpose, we constructed a special high-flow steam furnace, as described below. We have completed extensive equilibrium calculations on the stability of Ba(OH)_2_ liquids using available thermodynamic data, including the effect of CO_2_ contamination. Based on the calculated stabilities, we designed experiments using the high-flow steam furnace to test for the formation Ba(OH)_2_(liq) according to its predicted stability field. As a background for this work, differential thermal analysis (DTA) experiments were completed on carefully chosen compositions.

## 5. Experimental Procedure^1^

BaO was synthesized from 99.99 % purity (metals, by mass) BaCO_3_ by vacuum-calcining at 1300 °C for 10 h, followed by transfer to a glovebox. Complete decarbonation and conversion to BaO were verified by x-ray powder diffraction (XRD) in a sealed x-ray mount [16]. Powder XRD was completed on a Philips 2*θ* diffractometer^1^ using Cu K*_α_* radiation and a graphite monochromator. Diffractometer control and data acquisition were achieved using the JADE software system.

For melting studies, a Ba(OH)_2_/BaO mixture was prepared by controlled hydration of BaO. Melting and annealing experiments with simultaneous differential thermal analysis/thermogravimetric analysis (DTA/TGA) were completed in a Mettler TA1 thermoanalyzer outfitted with Anatech digital control and data acquisition electronics. DTA was completed at a ramp rate of 10 °C/min. The DTA apparatus was calibrated against the *α/β* quartz transition (571 °C) and the NaCl melting point (801 °C); DTA temperatures are estimated to have < ± 3 °C standard uncertainty. DTA crucibles were of dense slip-cast MgO.

**Fig. 3:**
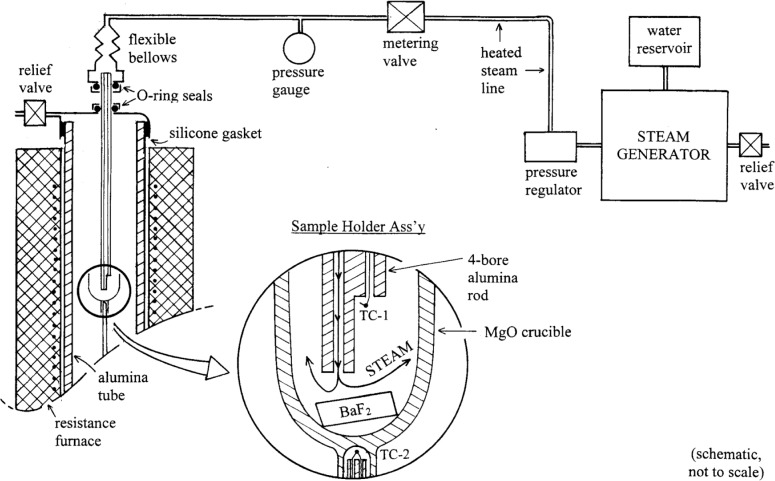
Schematic diagram of steam furnace set-up for generation of high $p_{H_{2}O}$ with rapid flow rates at elevated temperatures.

To enable experiments at high $p_{H_{2}O}$, a furnace capable of operation up to $p_{H_{2}O}=\left(0.1\text{MPa}\right)$ at temperatures above 800 °C was constructed, as shown schematically in Fig. 3. The special features of this furnace are: 1. a 3 kW steam generator provides an approximately constant-pressure source of steam for the furnace; 2. the steam generator uses a water feedstock with reduced CO_2_ (discussed below); 3. all areas of the furnace and supply lines are maintained at >100°C; 4. a type S thermocouple is positioned in the base of the sample crucible within 2 mm of the sample (TC-2); 5. the steam inlet is positioned in the crucible directly above the sample such that the steam flow must circulate around the sample before exiting the crucible; 6. steam flow can be reproducibly metered via a heated control valve; 7. a rapid flow of steam directly over the sample is possible. Steam furnace experimental results were examined optically using a Leica Wild M10 stereo-microscope and SPOT digital image capture software. Results were also characterized with an AMRAY 1400 scanning electron microscope (SEM) operated at ≈ 20 kV. Elemental compositions were determined with a Gresham energy dispersive x-ray spectrometer (EDS) and a 4Pi Analysis data acquisition interface controlled by Revolution software.

## 6. Calculative Procedure

Solid-liquid-gas equilibria in the system Ba(OH)_2_-BaO-BaF_2_-H_2_O-HF (and also with CO_2_) were calculated as a function of temperature using the Janaf thermochemical database [17] and the FactSage software suite [18]. The latter uses a Gibbs energy minimization algorithm to calculate equilibrium concentrations of species in the gas phase, and to calculate activities of liquid and solid phases. The calculative procedure involves minimizing the total stoichiometric summation of terms:

$$\begin{array}{r} G(i,T)=\left(\Delta_{f}H{{}^{\circ}}_{m}(i,298)+\int_{298}^{T}C_{p}(i)dT\right)\\ -T\left(S{}^{\circ}(i,298)+\int_{298}^{T}C_{p}(i)/TdT\right) \end{array}$$ (7)

for the collection of phases being considered, where *G*(i,*T*) = Gibbs energy function for phase i, ∆_f_ *H*°_m_(i, 298) = standard molar enthalpy of formation for phase i, *S* °(i, 298) = absolute molar entropy for phase i, *C*_p_(i) = molar heat capacity of phase i at constant pressure (0.1 MPa), and *T* = temperature kelvin. For solution phases, appropriate mixing terms must be added to Eq. (7), including terms of the form *RT*ln(*p*_j_), where *R* = gas constant and *p*_j_ = activity or partial pressure of species j. Species considered in the calculations are shown in Table 2. For the present calculations, gaseous species were considered to mix ideally. As described below, for purposes of calculation we assume there is negligible solubility of BaO and BaF_2_ in Ba(OH)_2_ liquids at the eutectic. Therefore to a first approximation the liquids are simple liquids, essentially pure. With regard to solid phases, there is evidence for solid solution of BaO in BaF_2_ [19], although a quantitative determination has not been made, and the extent is not known. Also, existence of a compound intermediate between BaF_2_ and Ba(OH)_2_ has been proposed [15]. As yet, there has been no definitive proof of such a compound, and there is no published x-ray diffraction pattern in the Powder Diffraction File [20]. However, it is clear that refinements in the calculations will need to be made, if additional data become available. For the present, the calculations give an approximate idea of the Ba(OH)_2_(liq) stability field. It is expected that solubility of BaF_2_ or BaO in Ba(OH)_2_(liq) would expand the liquid field of stability, whereas existence of an intermediate compound would have the opposite effect.

## 7. Effect of $p_{{\text{CO}}_{2}}$ on Ba(OH)_2_ Equilibria

One of the reasons for the relative success of the BaF_2_ *ex situ* method is the elimination of carbonate (usually present as BaCO_3_) from the processing route. Carbonate not only affects the kinetics of Ba_2_YCu_3_O_6.5 +_ *_x_*(s) formation due to the relative stability of BaCO_3_(s), but also affects the equilibrium phase assemblage by favoring formation of oxycarbonate phases such as Ba_4_Y_2_O_7_ · *x*CO_2_ and Ba_2_Y_2_O_5_ · *x*CO_2_ [21,22]. Formation of these phases interferes with Ba_2_YCu_3_O_6.5 +_ *_x_*(s) formation and complicates the decarbonation reactions which must take place for formation of Ba_2_YCu_3_O_6.5 +_ *_x_*(s) from BaCO_3_-containing precursors. Furthermore, presence of carbon in the superconductor phase has been shown to adversely affect properties [23].

**Fig. 4:**
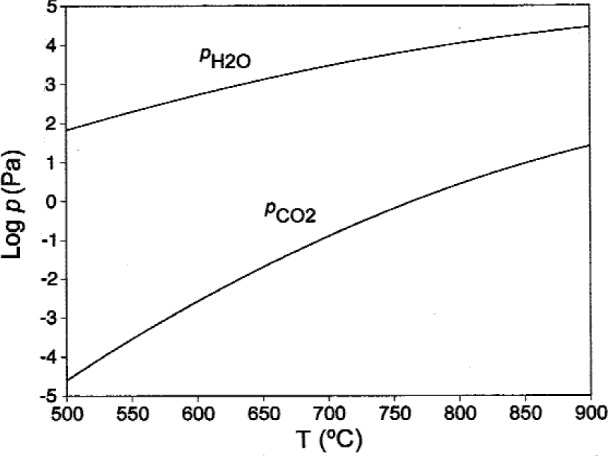
Equilibrium partial pressures of H_2_O and CO_2_ over coexisting Ba(OH)_2_ and BaCO_3_ as a function of temperature.

Calculations have been performed to estimate the effect of CO_2_ on Ba(OH)_2_ equilibria, by adding CO_2_(g), three polymorphs of BaCO_3_(s), and BaCO_3_(liq) to the list of species considered in Table 2. These calculations have been completed to determine the values of $p_{H_{2}O}$ and $p_{{\text{CO}}_{2}}$ which are in equilibrium with coexisting Ba(OH)_2_ and BaCO_3_, as shown in Fig. 4. From the curves in Fig. 4, at 500 °C, a $p_{{\text{CO}}_{2}}$ of < 10^−4.6^ Pa is required to avoid formation of BaCO_3_, while a $p_{H_{2}O}$ of > 10^1.8^ Pa) is necessary to stabilize Ba(OH)_2_. At 900 °C, $p_{{\text{CO}}_{2}}$ must be < 10^1.4^ Pa) to avoid formation of BaCO_3_, and $p_{H_{2}O}$ of > 10^4.5^ Pa is required to stabilize Ba(OH)_2_. For comparison, at 25 °C, water in equilibrium with air ( $p_{{\text{CO}}_{2}}=10^{1.5}\text{Pa}$) contains ≈ 10^−7^ mol fraction dissolved CO_2_. Water of this composition, if vaporized, would be within the BaCO_3_ stability field up to ≈ 625 °C. At 99 °C, water in equilibrium with air contains ≈ 10^−9^ mole fraction CO_2_, and if vaporized, would fall below the BaCO_3_ stability line above ≈ 525 °C. From these calculations it is clear that water used to generate $p_{H_{2}O}$ must be heated to reduce CO_2_ content if formation of BaCO_3_ is to be avoided. This is especially critical in experiments involving the formation of Ba(OH)_2_, such as those discussed below.

## 8. Ba(OH)_2_/BaO

The melting of a Ba(OH)_2_/BaO mixture was investigated by DTA to estimate the effect of BaO solubility on the melting temperature. A lowering of ≈ 3 °C was found, which lies within the estimated uncertainty of the DTA measurements. It is therefore concluded that the solubility of BaO in Ba(OH)_2_ liquids is negligible, and that Ba(OH)_2_ liquid can be modeled for calculational purposes as essentially pure Ba(OH)_2_.

**Fig. 5:**
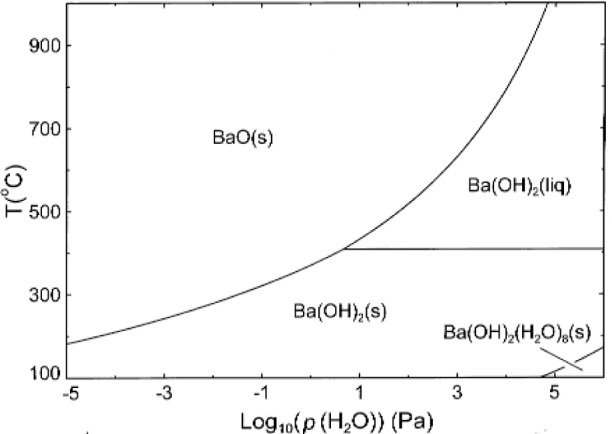
Log $p_{H_{2}O}$-T phase diagram of the system BaO-H_2_O calculated using available thermodynamic data [17, 18].

Accordingly, equilibria between solid and liquid Ba(OH)_2_ and BaO(s) were calculated and are presented in the phase diagram of Fig. 5. At $p_{H_{2}O}=0.1\text{MPa}$, Ba(OH)_2_(liq) is stable up to ≈ 1051 °C. At lower temperatures, the Ba(OH)_2_(s) stability field extends to very low values of $p_{H_{2}O}$ (< 10^−10^ Pa). The Ba(OH)_2_(liq) stability field is confined to a relatively narrow slice of the phase diagram above $p_{H_{2}O}\approx 10\text{Pa}$, lying between the phase fields of BaO(s) and Ba(OH)_2_(s). The most abundant gaseous species in equilibrium with Ba(OH)_2_(liq) along the Ba(OH)_2_(liq)/BaO(s) equilibrium curve are shown for selected temperatures in Table 3. While $p_{H_{2}O}$ is several orders of magnitude above the other partial pressures, it is noteworthy that at 900 °C, $p_{\text{Ba}{\left(\text{OH}\right)}_{2}}$ reaches ≈ 1 Pa. A partial pressure of this magnitude is sufficient to cause significant mass transport of Ba(OH)_2_ in the gas phase. At 25 °C (not shown in Fig. 5 or Table 3), the calculated $p_{H_{2}O}$ over coexisting Ba(OH)_2_(s) and BaO(s) is estimated to be 10^−14^ Pa, an indication of the difficulty in storing and handling BaO.

**Fig. 6:**
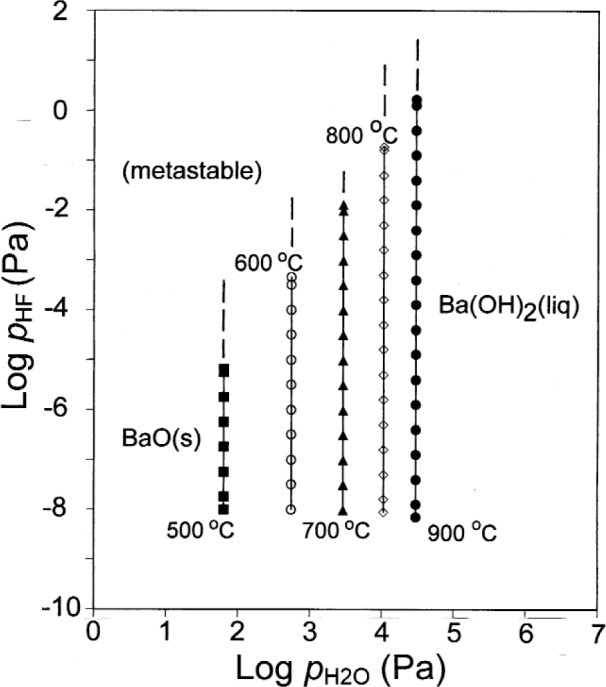
Ba(OH)_2_/BaO equilibrium as a function of log $p_{H_{2}O}$ and log *p*_HF_.

The effect of HF(g) *ex situ* conversion product on Ba(OH)_2_(liq)/BaO(s) equilibria must also be considered. As shown in Fig. 6, the log $p_{H_{2}O}$ values for the Ba(OH)_2_(liq)/BaO(s) equilibrium remain constant and independent of log *p*_HF_ for any given temperature. However the maximum value of log *p*_HF_ to which the Ba(OH)_2_(liq)/BaO(s) equilibrium is stable increases with increasing temperature. Above this value, indicated by the dashed parts of the equilibrium lines at any given temperature, the Ba(OH)_2_(liq)/BaO(s) equilibrium is metastable. Gas phase compositions for selected points near the centers of the equilibrium boundaries in Fig. 6 are given in Table 4. Comparison of Table 3 and Table 4 reaffirms the constancy of the equilibrium $p_{H_{2}O}$ in the presence of HF.

## 9. Ba(OH)_2_/BaF_2_

Due to the formation of Ba(OH)_2_-hydrates, it was not possible to prepare pure Ba(OH)_2_ from BaO during this investigation, and therefore the melting point lowering of Ba(OH)_2_/BaF_2_ mixtures was not investigated experimentally. For calculational purposes it is therefore assumed that the solubility of BaF_2_ in Ba(OH)_2_(liq) at the eutectic is small, a reasonable assumption, given the much higher melting point of BaF_2_ (> 1300 °C).

**Fig. 7:**
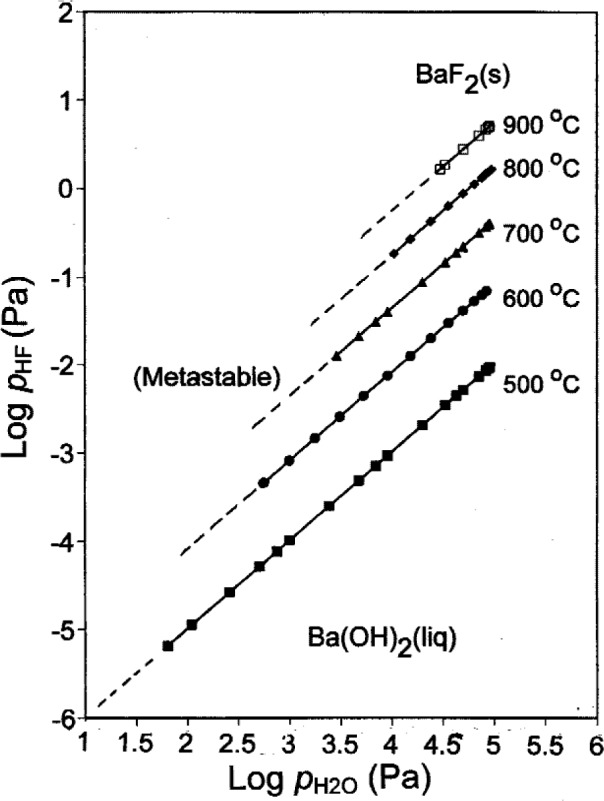
Ba(OH)_2_/BaF_2_ equilibrium as a function of log $p_{H_{2}O}$ and log *p*_HF_.

To describe the equilibrium between Ba(OH)_2_(liq) and BaF_2_(s), it is necessary to consider *p*_HF_ as well as $p_{H_{2}O}$. Figure 7 shows calculated curves for the Ba(OH)_2_(liq)/BaF_2_(s) equilibrium at several temperatures. As the temperature increases, the maximum *p*_HF_ value to which the Ba(OH)_2_(liq) stability field extends increases. As temperature increases, the minimum $p_{H_{2}O}$ value to which the Ba(OH)_2_(liq) stability field extends also increases. Below this value, the Ba(OH)_2_(liq)/BaF_2_(s) equilibrium is metastable. Gas phase compositions at selected points on the curves in Fig. 7 are given in Table 5. At higher temperatures, as for the Ba(OH)_2_(liq)/BaO(s) equilibrium, the values of $p_{\text{Ba}{\left(\text{OH}\right)}_{2}}$ become significant. From the slopes of Fig. 7 and the data in Table 5, a given increase in $p_{H_{2}O}$ results in a proportionately much larger increase in the equilibrium *p*_HF_. For example, an order-of-magnitude increase in $p_{H_{2}O}$ produces a two orders-of-magnitude increase in *p*_HF_. The requirements for HF(g) removal during the conversion of BaF_2_(s) to Ba(OH)_2_(liq) are significantly reduced by maintaining increased $p_{H_{2}O}$, as well as by maintaining higher temperatures.

## 10. Ba(OH)_2_/BaO/BaF_2_

**Fig. 8:**
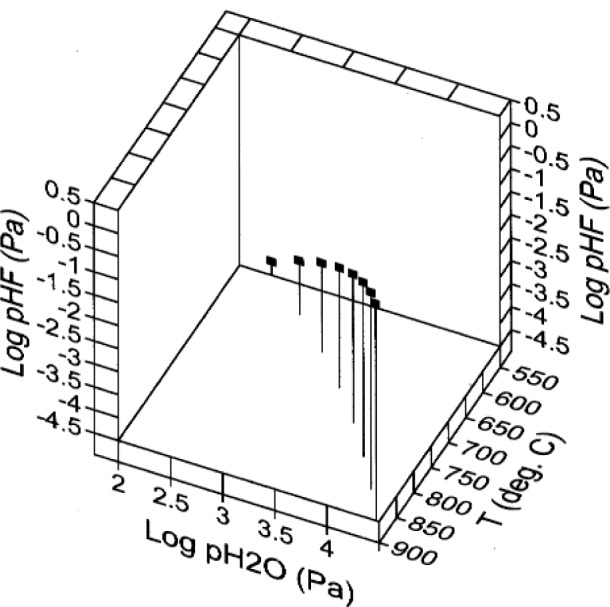
Three-phase Ba(OH)_2_/BaO/BaF_2_ equilibrium as a function of log $p_{H_{2}O}$, log *p*_HF_, and temperature. Dashed lines indicate orthogonal projection of the equilibrium curve onto the sidewalls of the 3-D plot.

Treatment of equilibria involving the three phases Ba(OH)_2_, BaO, and BaF_2_ is facilitated by the use of the 3-D plot shown in Fig. 8, where the three-phase Ba(OH)_2_/BaO/BaF_2_ equilibrium is represented as a function of $p_{H_{2}O}$, *p*_HF_, and temperature. The three-phase equilibrium is actually a curve in log $p_{H_{2}O}$ - log *p*_HF_ -temperature parameter space, as illustrated by its projection onto the base of the plot. Table 6 gives gas phase compositions at selected points along the three-phase equilibrium.

**Fig. 9:**
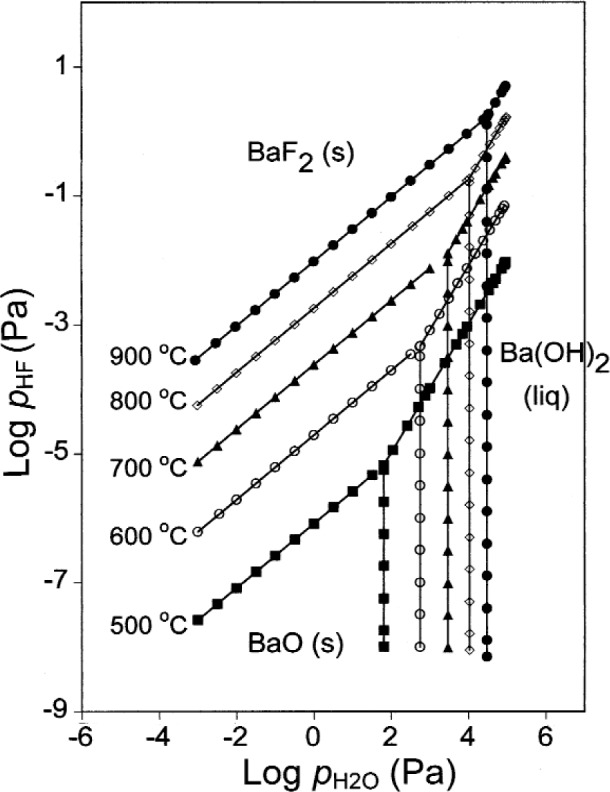
Isothermal sections through three-phase Ba(OH)_2_/BaO/BaF_2_ region in Fig. 8.

The data in Fig. 8 can also be conveniently represented through the use of isothermal sections, as shown in Fig. 9. Here the three-phase Ba(OH)_2_/BaO/BaF_2_ equilibrium plots as a point at the juncture of the areas corresponding to the Ba(OH)_2_, BaO, and BaF_2_ stability fields. With increasing temperature, the stability field of Ba(OH)_2_ shrinks to higher $p_{H_{2}O}$, but simultaneously expands to higher *p*_HF_.

On the basis of Fig. 9, a steam furnace experiment was designed to test for the formation of Ba(OH)_2_(liq) from BaF_2_(s). Using the steam furnace, $p_{H_{2}O}$ of 0.1 MPa at temperatures of 800 °C or above can be maintained for extended periods of time. These are the optimum conditions for formation of Ba(OH)_2_(liq) according to reaction (5), provided *p*_HF_ can be maintained at < 1 Pa. In practice, the latter requirement can be met by rapidly flowing steam over the sample to remove product HF, thereby reducing *p*_HF_ to low levels.

Results of a steam furnace experiment in which a single crystal fragment of optical quality BaF_2_ was held at 815 °C, for 2h, with $p_{H_{2}O}=0.1\text{MPa}$, with a steam flow over the sample estimated at > 0.2 L/s, are shown in Fig. 10a. Fluorine on the surface of the BaF_2_ has been uniformly replaced by oxygen to a depth of at least 1 µm, as estimated by the lack of a fluorine EDS signal from the underlying BaF_2_ (Fig. 10b). The full EDS spectrum (not presented) shows Ba and O as the main constituents on the surface of the reacted crystal, with a relatively small C K_α_ peak (no method was available for detection of hydrogen). The smooth, dense nature of the reacted surface is consistent with the formation of Ba(OH)_2_ liquid. As discussed above, CO_2_ in the water feedstock for this experiment was reduced to low levels such that the formation of BaCO_3_ was minimized. The EDS spectrum of Fig. 10b indicates that BaCO_3_ was not a major reaction product, as the C K_α_ intensity is similarly low for both reacted and unreacted crystals. The presence of C in both spectra is an indication of minor hydrocarbon surface contamination. Smaller peaks present in the reacted sample are due to trace contaminants from the steam boiler and transport line (Si, possibly Co), and are not likely to have had a significant effect on the F/O reaction. We conclude that formation of Ba(OH)_2_(liq) from BaF_2_(s) according to reaction (5) is the most probable explanation of the results in Fig. 10.

The high-flow experiment at 815 °C, $p_{H_{2}O}=0.1\text{MPa}$ may be near the upper limit of conditions useful for practical processing of second-generation coated conductors, due to the thermal limitations of currently available substrate/buffer combinations. From Fig. 9, production of Ba(OH)_2_ liquids at lower temperatures requires more complete removal of HF from the reaction site. An estimate of the requirements for Ba(OH)_2_(liq) formation according to reaction (5) at 700 °C can be made as follows. First, it must be noted that, at 700 °C, *p*_HF_ must be ≈< 10^−1^ Pa for Ba(OH)_2_(liq) to form. At a steam flow rate of 0.2 L/s over the sample, this gives a maximum HF removal of 10^−3.8^ mL/s from the reaction site, assuming equilibrium. The resulting rate of formation of Ba(OH)(liq) is 10^−8.1^ mol/s. If reaction (5) is the rate-limiting step, then Ba_2_YCu_3_O_6.5 +_ *_x_* would be formed by reaction (6) at a rate of ≈ 10^−8.4^mol/s. For a 1 cm^2^ area, this corresponds to a thickness conversion rate of ≈ 10^−2.3^ µm/s at 700 °C. Thus, formation of Ba_2_YCu_3_O_6.5 +_ *_x_* superconducting films of 1 µm thickness over an area of 1 cm^2^ could conceivably be achieved in 200 s to 250 s, with potential for scale-up to much larger production rates. High-performance films with 1 µm or greater superconductor thickness could find immediate application in second-generation high *T*_c_ technology, especially if production costs approach the anticipated $10/kA-m target [24].

## 11. Summary and Conclusions

Thermodynamic calculations have outlined a stability field for Ba(OH)_2_(liq) as a function of $p_{H_{2}O}$, *p*_HF_, and temperature, based on presently available data. An experiment at 815 °C, $p_{H_{2}O}=0.1\text{MPa}$ has provided evidence for the formation of Ba(OH)_2_(liq) by defluorination of BaF_2_(s), as predicted by the calculations. It is possible that under conditions of high $p_{H_{2}O}$ and rapid gas flow, the formation of Ba(OH)_2_(liq) may occur as an intermediate step in the formation of superconducting Ba_2_YCu_3_O_6.5 +_ *_x_*(s) from amorphous (BaF_2_, Y, Cu) precursors. The presence of a Ba(OH)_2_ liquid could be important for Ba_2_YCu_3_O_6.5 +_ *_x_*(s) processing for several reasons. It is well known that a liquid phase enhances mobility and can aid in local mass transport and the development of oriented microstructures. The presence of a liquid would be expected to improve the kinetics of Ba_2_YCu_3_O_6.5 +_ *_x_*(s) phase formation, although it may be necessary to limit the presence of the liquid phase at some stages during processing to prevent random growth, as opposed to oriented growth. Clearly, it is essential to control the amount of liquid in order to fully optimize all aspects of Ba_2_YCu_3_O_6.5 +_ *_x_*(s) formation using the BaF_2_ *ex situ* process. With sufficient data on liquid formation, $p_{H_{2}O}$ provides an additional parameter, along with precursor F/O composition, gas flow, temperature, time, and $p_{O_{2}}$, with which to reproducibly control the processing of Ba_2_YCu_3_O_6.5 +_ *_x_*(s).

The calculated phase diagrams require further experimental verification to establish the role of BaF_2_ solubility in the liquid, and the precise boundaries of the Ba(OH)_2_ stability field. Preliminary experiments have indicated that Ba_2_YCu_3_O_6.5 +_ *_x_*(s) is stable in the presence of Ba(OH)_2_(liq) [25]; it is essential to know the range of conditions under which the Ba_2_YCu_3_O_6.5 +_ *_x_*(s) and the Ba(OH)_2_ stability fields overlap. Based on the extent to which Ba(OH)_2_-based liquids extend into the phase space of Fig. 2, it may prove possible to design processing routes to control intersection of the PVD and MOD processing paths with the hydroxide liquid phase field. Then the full range of $p_{H_{2}O}$-temperature processing space can be explored to determine if there are new processing routes which might lead to further optimization of superconductor formation and film properties.

## Figures and Tables

**Fig. 2a:**
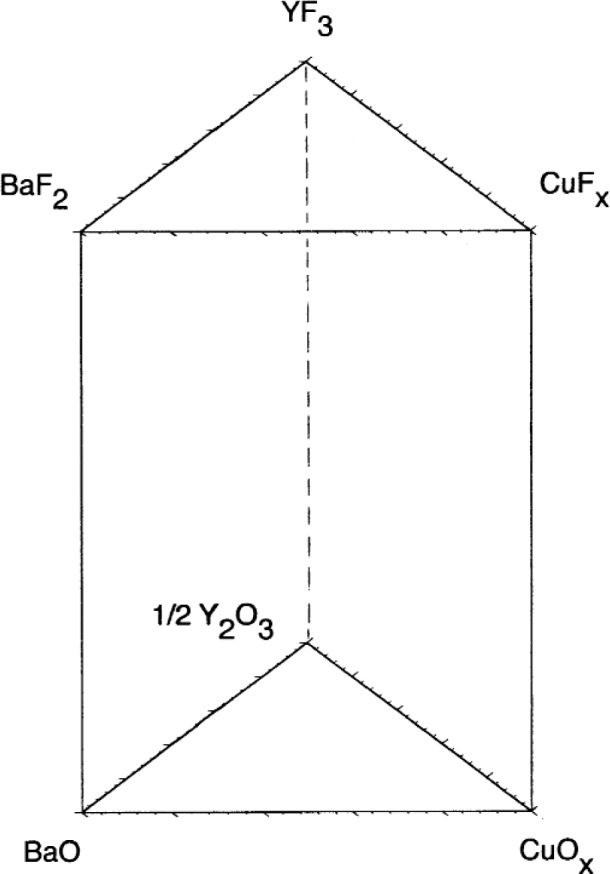
Trigonal prism used for compositional model of BaF_2_ *ex situ* phase equilibria.

**Fig. 2b:**
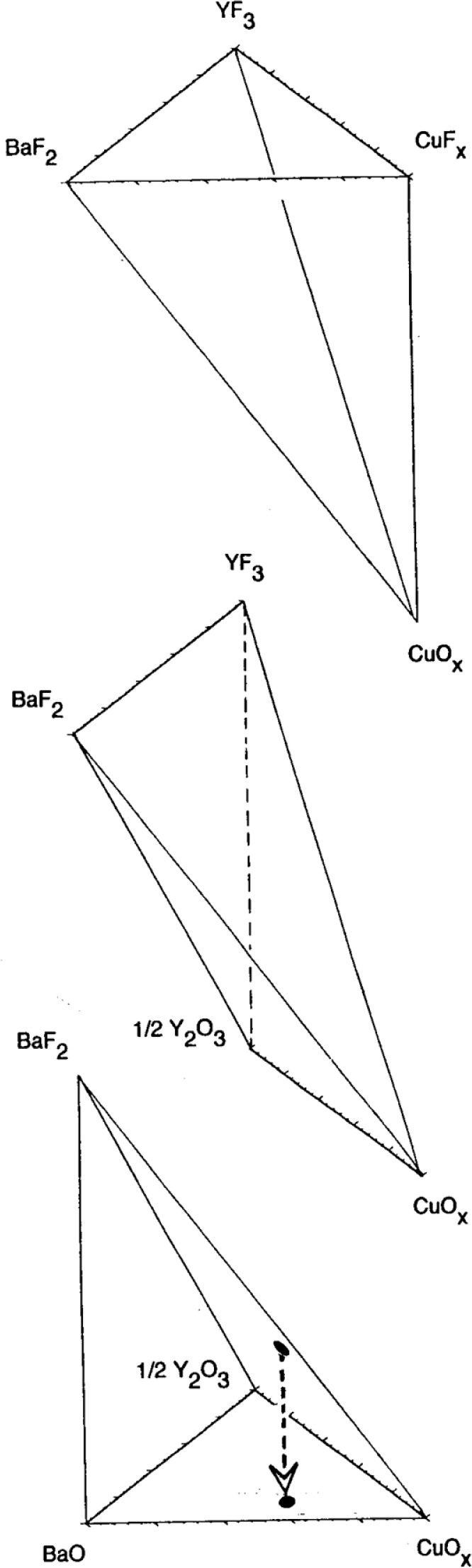
Phase compatibility tetrahedra comprising the trigonal prism. Idealized *ex situ* processing path for PVD films is shown in bottom tetrahedron.

**Fig. 10a:**
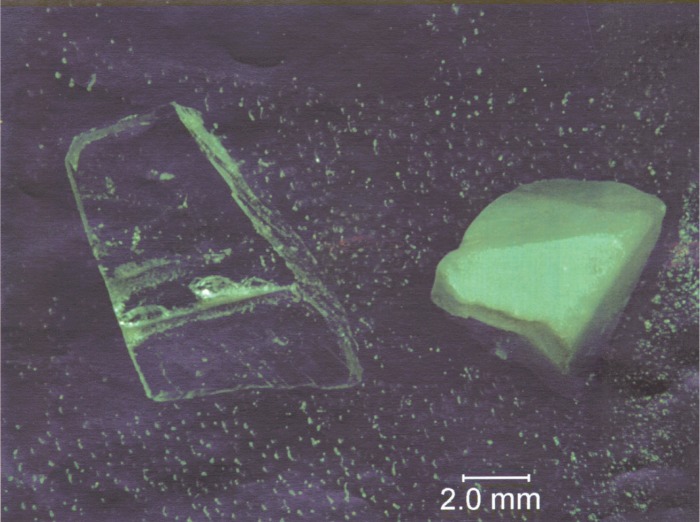
Results of BaF_2_ single crystal placed in steam furnace at 815 °C, 2 h, $p_{H_{2}O}=0.1\text{MPa}$. Clear crystal on left is unreacted. Reaction produced dense white opaque coating on crystal at right.

**Fig. 10b:**
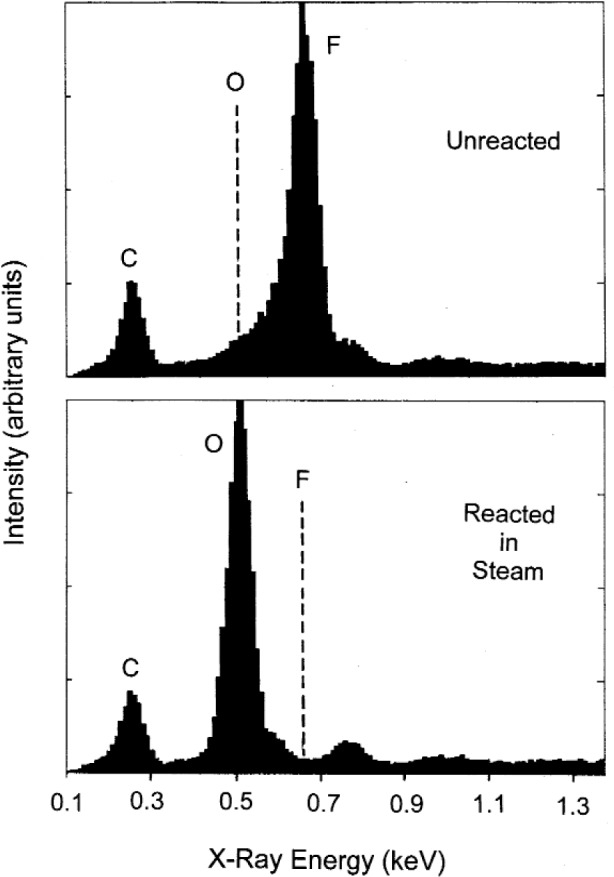
Comparative EDS spectra from samples in (a) show that reaction has resulted in essentially complete replacement of F by O at the surface.

**Table 1:** Relative thermodynamic stabilities in the system Ba, Y, Cu//O. F at 500 °C, 0.1 MPa (reactants and products in most stable form)

Reactants		Products	Gibbs energy of reaction
3 CuF_2_ + Y_2_O_3_	→	3 CuO + 2 YF_3_	−396 kJ
2 YF_3_ + 3 BaO	→	3 BaF_2_ + Y_2_O_3_	−413 kJ
CuF_2_ + BaO	→	BaF_2_ + CuO	−270 kJ

**Table 2:** Gaseous species and condensed phases included in Ba(OH)_2_/BaO/BaF_2_ equilibrium calculations

Gaseous species	Solids	Liquids
H,H_2_,O,O_2_,O_3_,OH,H_2_O,HOO, HOOH, F, F_2_, HF, (HF)_2_, H_3_F_3_, H_4_F_4_, H_5_F_5_, H_6_F_6_, H_7_F_7_, OF, O_2_F, HOF, Ba, Ba_2_, BaH, BaH_2_, BaO, Ba_2_O, Ba_2_O_2_, BaOH, Ba(OH)_2_, BaF, BaF_2_,	BaF_2_, BaO, Ba(OH)_2_, Ba(OH)_2_⋅8H_2_O	Ba, BaO, Ba(OH)_2_ BaF_2_

**Table 3:** Gas phase partial pressures (Pa) in equilibrium with Ba(OH)_2_(liq)/BaO(s)

*T*(°C)	$p_{H_{2}O}$	$p_{H_{2}}$	$p_{\text{Ba}{\left(\text{OH}\right)}_{2}}$	$p_{O_{2}}$	*p*_OH_
500	10^1.8^	10^−6.3^	10^−6.4^	10^−6.6^	10^−8.2^
600	10^2.7^	10^−4.4^	10^−4.2^	10^−4.7^	10^−6.0^
700	10^3.5^	10^−2.8^	10^−2.5^	10^−3.2^	10^−4.3^
800	10^4.0^	10^−1.7^	10^−1.2^	10^−2.0^	10^−2.9^
900	10^4.5^	10^−0.7^	10^−0.1^	10^−1.0^	10^−1.8^

**Table 4:** Gas phase partial pressures (Pa) in equilibrium with Ba(OH)_2_(liq)/BaO(s) in the presence of HF

*T*(°C)	$p_{H_{2}O}$	*p*_HF_	$p_{H_{2}}$	$p_{\text{Ba}{\left(\text{OH}\right)}_{2}}$	$p_{O_{2}}$	*p*_OH_
500	10^1.8^	10^−6.3^	10^−6.3^	10^−6.4^	10^−6.6^	10^−8.2^
600	10^2.7^	10^−5.5^	10^−4.4^	10^−4.3^	10^−4.7^	10^−6.0^
700	10^3.5^	10^−5.0^	10^−2.9^	10^−2.5^	10^−3.2^	10^−4.3^
800	10^4.0^	10^−4.3^	10^−1.7^	10^−1.2^	10^−2.0^	10^−2.9^
900	10^4.5^	10^−3.9^	10^−1.7^	10^−0.1^	10^−1.0^	10^−1.3^

**Table 5:** Gas phase partial pressures (Pa) in equilibrium with Ba(OH)_2_(liq)/BaF_2_(s)

*T*(°C)	$p_{H_{2}O}$	*p*_HF_	$p_{H_{2}}$	$p_{\text{Ba}{\left(\text{OH}\right)}_{2}}$	$p_{O_{2}}$	*p*_OH_
500	10^3.4^	10^−3.6^	10^−5.2^	10^−6.4^	10^−5.5^	10^−7.2^
600	10^4.0^	10^−2.1^	10^−3.6^	10^−4.2^	10^−3.9^	10^−5.2^
700	10^4.3^	10^−1.1^	10^−2.3^	10^−2.5^	10^−2.6^	10^−3.7^
800	10^4.6^	10^−0.2^	10^−1.3^	10^−1.2^	10^−1.6^	10^−2.6^
900	10^4.7^	10^0.4^	10^−0.6^	10^−0.1^	10^−0.8^	10^−1.6^

**Table 6:** Gas phase partial pressures (Pa) in equilibrium with Ba(OH)_2_(liq)/BaO(s)/BaF_2_(s)

*T*(°C)	$p_{H_{2}O}$	*p*_HF_	$p_{H_{2}}$	$p_{\text{Ba}{\left(\text{OH}\right)}_{2}}$	$p_{O_{2}}$	*p*_OH_
500	10^1.8^	10^−5.2^	10^−6.3^	10^−6.4^	10^−6.6^	10^−8.2^
600	10^2.7^	10^−3.3^	10^−4.4^	10^−4.2^	10^−4.7^	10^−6.0^
700	10^3.5^	10^−5.0^	10^−2.9^	10^−2.5^	10^−3.2^	10^−4.3^
800	10^4.0^	10^−4.3^	10^−1.7^	10^−1.2^	10^−2.0^	10^−2.9^
900	10^4.5^	10^−3.9^	10^−1.7^	10^−0.1^	10^−1.0^	10^−1.3^

## Footnotes

1 Certain commercial equipment, instruments, or materials are identified in this paper to foster understanding. Such identification does not imply recommendation or endorsement by the National Institute of Standards and Technology, nor does it imply that the materials or equipment identified are necessarily the best available for the purpose.
